# Effectiveness of eHealth Interventions and Information Needs in Palliative Care: A Systematic Literature Review

**DOI:** 10.2196/jmir.2812

**Published:** 2014-03-07

**Authors:** Daniel Capurro, Matthias Ganzinger, Jose Perez-Lu, Petra Knaup

**Affiliations:** ^1^Evidence Based Health Care Program, Department of Internal MedicineEscuela de MedicinaPontificia Universidad Católica de ChileSantiagoChile; ^2^Department of Biomedical Informatics and Medical EducationUniversity of WashingtonSeattle, WAUnited States; ^3^Institute of Medical Biometry and InformaticsUniversity of HeidelbergHeidelbergGermany; ^4^School of Public Health and AdministrationUniversidad Peruana Cayetano HerediaLimaPeru

**Keywords:** palliative Care, eHealth, systematic review

## Abstract

**Background:**

One of the key components in palliative care is communication. eHealth technologies can be an effective way to support communications among participants in the process of palliative care. However, it is unclear to what extent information technology has been established in this field.

**Objective:**

Our goal was to systematically identify studies and analyze the effectiveness of eHealth interventions in palliative care and the information needs of people involved in the palliative care process.

**Methods:**

We conducted a systematic literature search using PubMed, Embase, and LILACS according to the Preferred Reporting Items for Systematic Reviews and Meta-Analyses (PRISMA) guidelines. We collected and analyzed quantitative and qualitative data regarding effectiveness of eHealth interventions and users’ information needs in palliative care.

**Results:**

Our search returned a total of 240 articles, 17 of which met our inclusion criteria. We found no randomized controlled trial studying the effects of eHealth interventions in palliative care. Studies tended to be observational, noncontrolled studies, and a few quasi-experimental studies. Overall there was great heterogeneity in the types of interventions and outcome assessments; some studies reported some improvement on quality of care, documentation effort, cost, and communications. The most frequently reported information need concerned pain management.

**Conclusions:**

There is limited evidence around the effectiveness of eHealth interventions for palliative care patients, caregivers, and health care professionals. Focused research on information needs and high-quality clinical trials to assess their effectiveness are needed.

## Introduction

### Background

 The World Health Organization (WHO) defines palliative care as “an approach that improves the quality of life of patients and their families facing the problem associated with life-threatening illness, through the prevention and relief of suffering” [1]. Further, WHO clearly states that implementing “a support system to help patients live as actively as possible until death” [1] is one of the responsibilities of palliative care. In the WHO’s definition, the aforementioned support system does not necessarily refer to a technical system, but to all the infrastructure and supporting services that are needed to help patients in palliative care, which may include information technologies (IT).

As the world population grows older, chronic noncommunicable conditions have emerged as the main causes of mortality worldwide. It is estimated that there are 600 million people older than 60 years, and of the 58 million deaths that occur each year, 60% can be attributed to progressive conditions such as cancer and cardiovascular diseases [2]. These progressive conditions determine an increasing demand for palliative care. Considering this large and ever-growing demand, as well as the scarcity of trained specialists in palliative care to meet those needs, both in developed and developing countries, it is critical to identify methods to augment available resources. Given the scalability of tools and interventions based on information technologies, we believe that there is a high potential for IT tools and applications in palliative care.

### eHealth

eHealth has been defined as “health services and information delivered or enhanced through the Internet and related technologies” [3], and it has been progressively incorporated into the options available to deliver health care. In other domains of health care, eHealth has been successfully used to support patients and clinicians during the routine tasks involved in clinical care. Among many examples, we can find a study conducted by Clarke et al [4] that demonstrated that patients allocated to receive an Internet self-help intervention had a greater reduction in depressive symptoms than the control group. Similarly, another study tested the use of SMS text messaging (short message service) to monitor low back pain in primary care patients [5].

Considering the key role of information and communication in palliative care, these positive effects might also be true for patients, family members, and clinicians in the domain of palliative care. To validate this assumption, we identified two broad questions to guide this research:

(1) What eHealth interventions with proven efficacy exist for use in palliative care? It is unclear whether the advances eHealth made in recent years are already available in the area of palliative care. Further, the efficacy of such measures remains uncertain.

(2) What is known about the information needs of the participants in the palliative care process? To be efficient and successful, an existing or planned eHealth system needs to fulfill these specific information needs.

### eHealth for Palliative Care

Several attempts have been made to extend the coverage of currently available resources in palliative care. A traditional communication technology frequently used with this purpose in palliative care is the telephone. Several studies have reported on the usefulness of phone services to support palliative care patients and caregivers. However, since telephone communications require the synchronous interaction between the communicating parties, it is expensive to scale them up since they require proportional availability of human resources. Today, with the advances of information and communication technologies (the Internet, mobile phones, and smartphone applications), there may be additional evidence about the effectiveness of nonsynchronous communications.

To our knowledge, no systematic review of the research conducted in this field has been published. The goal of this study was to conduct a systematic literature review with the following two objectives: (1) identify studies on eHealth interventions in palliative care that assessed the efficacy of the intervention, and (2) identify studies reporting on the information needs of patients, caregivers, and health professionals in palliative care.

The identified studies will be used to identify current knowledge gaps and to highlight the areas where more research is needed regarding the use of IT to support palliative care. Once the information needs are known, it will be possible to develop eHealth systems adequately tailored to meet those needs.

## Methods

### Eligibility Criteria

#### Inclusion Criteria

For this systematic literature review, we followed the Preferred Reporting Items for Systematic Reviews and Meta-analyses (PRISMA) guidelines [6]. To assess the effectiveness of eHealth interventions, we included experimental or observational studies published in English, German, or Spanish that assessed the effectiveness of eHealth interventions for patients in palliative care or those involved in their care such as caregivers or health care providers. We defined eHealth interventions as any information and communication technology designed to conduct measurements, enhance communications, or deliver relevant information for patients, caregivers, or health care providers. We excluded communication technologies that relied exclusively on synchronous communication (eg, telephone or video consultations), given the limited scalability of such systems (ie, a health professional can answer only one call at a time). If the number of patients rises, callers may have to wait in a queue or the number of health professionals must be increased to take calls in parallel. We did include systems based on mobile phones when they consisted of asynchronous communications such as SMS text messaging (short message service) or smartphone applications.

To understand users’ information needs, we included experimental or observational studies published in English, German, or Spanish that explicitly assessed the information needs related to eHealth applications of patients in palliative care or those involved in their care, such as caregivers or health care providers. We defined eHealth interventions as any information and communication technology designed to conduct measurements, enhance communications, or deliver relevant information for patients, caregivers, or health care providers, including synchronous communication like telephone or video consultations. We accepted reports on systems based on synchronous communication for the search on users’ needs because these studies might provide important information for the future development of an asynchronous eHealth system as defined above.

#### Exclusion Criteria

Reports that met the following criteria were excluded from this study: articles not assessing the effectiveness of eHealth applications or participant’s information needs; articles reporting on interventions with synchronous communication; articles not involving palliative care patients, caregivers, or health care providers; letters, editorials, white papers, and conference abstracts; and articles written in languages other than English, German, or Spanish.

### Search Strategy

To identify relevant studies, we conducted electronic searches in June 2012 using PubMed, Embase, and LILACS (Literature in the Health Sciences in Latin America and the Caribbean). We constructed a search strategy using the following terms, adapted to each of the databases used, using standard terminology when available: (smartphone OR “Short Message Service” OR “SMS” OR “text messaging” OR “text message” OR “Cellular Phone” OR “Electronic Medical Record” OR “Patient Health Record” OR “Telemedicine” OR “mhealth” OR “m-health” OR “ehealth” OR “e-health” OR “mobile health” OR “electronic health”) AND (“Palliative Care”).

### Study Selection and Data Extraction

To assess whether they met our inclusion and exclusion criteria, 2 researchers independently reviewed the titles and abstracts of all retrieved articles. Disagreement was resolved by consensus. If the title and abstract were insufficient to decide the inclusion, we reviewed the article’s full text to decide on final inclusion or exclusion. In addition to the electronic search, we manually reviewed citations of relevant publications identified through the electronic search. Once a final list of articles was available, we obtained the full text of all articles for complete data extraction.

Relevant data were extracted from all included articles using an online data capture form. To increase the consistency of data extraction, three researchers conducted an initial parallel data extraction of a subset of articles. The results were reviewed and disagreements resolved. The process was further repeated until consistent data extraction was obtained. Two researchers extracted the data of the remaining articles independently. Some additional articles were excluded during the data extraction stage if the abstract presented misleading information about the scope of the study.

## Results

### Summary

**Figure 1:**
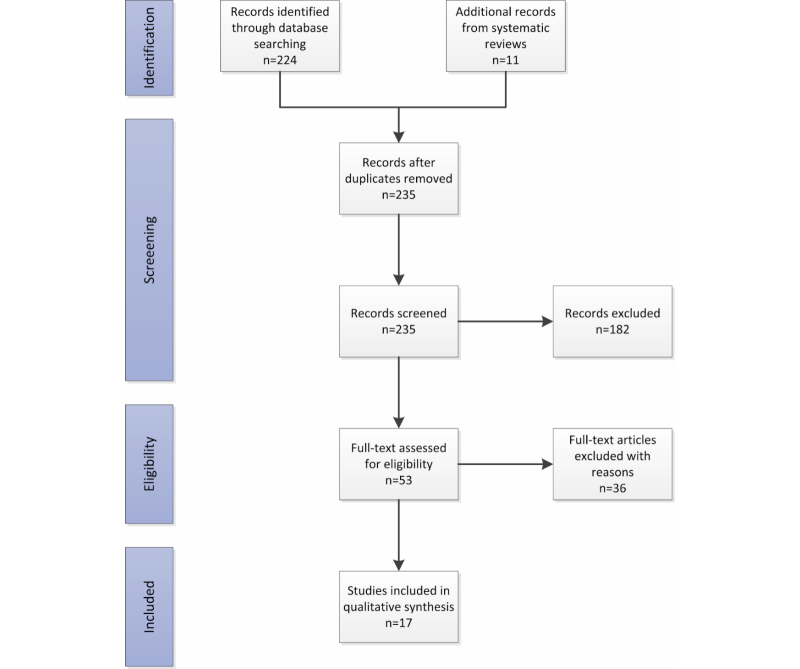
PRISMA flowchart of the systematic review.

The flowchart depicting the results of the literature selection process is shown in Figure 1. Our database queries resulted in 237 records. Two of these results were systematic literature reviews [7,8] from which we decided to manually review their citations. They included 11 additional research papers not included in the original database search. These papers were added to our review process. After screening titles and abstracts, we excluded 187 papers from our data pool. Assessment of the remaining papers’ full texts resulted in the exclusion of another 36 papers since they did not meet our inclusion criteria. The respective reasons for exclusion are reported in Multimedia Appendix 1. All resulting papers were written in English. In the end, 16 publications were identified through the database search [9-24], and one additional article was identified by manually reviewing citations from relevant publications [25].

### Key Feature of Selected Studies

Table 1 shows an overview of the 17 included papers. In terms of location and resources available, all but 1 study were conducted in high-income countries, using the World Bank classification [26]; 7 studies were conducted in Europe, 6 in North America, 3 in Australia, and only 1 study was done in Africa. The 7 studies included participants from rural settings, 2 from urban settings, and the rest did not provide specific information in that regard. With respect to the types of the studies, 11 were observational studies, 3 quasi-experimental, 1 was a hypothesis-generating study, and 1 was a narrative case study. There was wide variability on information about units of analysis and sample sizes. For example, some studies measured individual visits or patient numbers; others considered single consultations or phone calls. The follow-up time of the studies could be extracted only from 3 papers and ranged between 2 weeks and 1229 hours.

As far as our study questions are concerned, two of the papers cover both of our questions. Users’ information needs in palliative care were described in nine publications, while six publications assessed only the efficacy of eHealth systems for palliative care.

Table 2 summarizes the types of interventions assessed by the included studies. The most frequent intervention was the implementation of telephone-based support infrastructures. Other interventions aimed to simplify various aspects of documentation, for example, by introducing digital pens or by simplifying the use of questionnaires with eHealth methods (eg, [27]). The wide variations in study methods, interventions, and outcomes assessed prevented any attempt to pool results.

Table 3 summarizes the participants of the studies and the number of occurrences in the studies included. A study could have participants of more than one type.

**Table 1:** Overview of the studies included in the systematic review.

Author, year	Intervention	Location	Type of study	Sample size	Follow-up
Coleman 2005 [25]	Study about the effects of a frequently asked questions module added to an Internet chat room on pancreatic cancer	USA (Internet users)	Quasi-experimental (nonrandomized controlled trials, time series, before-after)	600 posts	N/A
Grant 2011 [9]	Qualitative assessment of 3 palliative care programs in extremely underserved areas; qualitative findings included the utility of mobile phones to communicate with the palliative care team	Uganda (rural), Kenya (rural), Malawi (peri-urban)	Observational: Cross-sectional	3 programs	N/A
Kallen 2012 [10]	Improve patient symptom status reporting and symptom management with novel software	Texas, United States (setting unclear)	Observational: Cross-sectional	27	N/A
Koczwara 2010 [11]	An education program for rural health providers incorporating information on palliative cancer treatments and supportive care, as well as strategies on how to provide effective multidisciplinary care in rural settings	Australia (rural)	Observational: Cross-sectional	90	1229 hours
Lind 2004 [12]	Development and assessment of system to monitor patient symptoms using digital pens	Linköping, Sweden (setting unclear)	Observational: Cross-sectional	12	N/A
Lind 2008 [13]	Qualitative study assessing the impact of a digital pen–based system to monitor symptoms	Linköping, Sweden (setting unclear)	Observational: Cross-sectional	12	2 weeks
Linklater 2009 [14]	Evaluation of a specialist palliative care helpline for general practitioners	Grampian, United Kingdom (rural)	Observational: Cross-sectional	1146 calls	N/A
Maudlin 2006 [15]	Pilot study for using specialized teams and technology to enhance care and support veteran patients and their families	Florida/Puerto Rico, United States	Quasi-experimental (nonrandomized controlled trials, time series, before-after)	100	N/A
McCall 2008 [16]	Study testing the feasibility of using mobile phone–based technology to monitor and manage symptoms reported by patients being cared for at home in the advanced stages of their illness	Scotland, United Kingdom (rural)	Observational: Cross-sectional	21 patients, 9 HC professionals	30 days
Van Heest 2009 [17]	Analysis of surveys conducted after palliative care consultations between GPs and palliative care specialists	The Netherlands (rural)	Observational: Cross-sectional	415 consultations	N/A
Paré 2009 [18]	Evaluation of effects of a provider-focused telehomecare intervention implemented in an oncology and palliative care unit	Quebec, Canada (urban)	Quasi-experimental (nonrandomized controlled trials, time series, before-after)	7	N/A
Phillips 2008 [19]	Qualitative assessment of a telephone consultation service.	New South Wales, Australia (rural)	Other hypothesis- generating studies (eg, qualitative inquiry)	8 caregivers, unclear number of health care professionals	N/A
Ridley 2008 [20]	Study examines a 24-hr telephone hotline available to physicians, nurses, and pharmacists across the province.	British Columbia, Canada (rural and urban)	Other hypothesis- generating studies (eg, qualitative inquiry)	692 calls	N/A
Roberts 2007 [21]	Feature on telephone support for palliative care patients and their carers	Canada (setting unclear)	Report	254	N/A
Teunissen 2007 [22]	Descriptive assessment of a telephone consultation system	Utrecht, Netherlands (setting unclear)	Observational: Cross-sectional	2089 consultations	N/A
Walker 2006 [23]	Evaluation of an electronic tool that can be used on PDAs for assessment of patient-reported outcomes (patient’s symptoms)	St. Gallen, Switzerland (setting unclear)	Observational: Cross-sectional	54 patients	N/A
Wilkes 2004 [24]	Descriptive evaluation using an audit of telephone logbook, text analysis of reflective journals, questionnaire, and interviews	Grafton, New South Wales, Australia (rural)	Observational: Cross-sectional	69	N/A

**Table 2:** Summary of interventions described in included papers.

Category of intervention	Number of articles	Reference
Education	1	[11]
Other	3	[9,17,25]
Digital pen	2	[12,13]
Phone support	6	[14,19-22,24]
Process software	2	[10,18]
Questionnaire, phone	1	[16]
Questionnaire, other	2	[15,23]

**Table 3:** Types of participants covered by the studies.

Participants	Number of studies
Patients	11
Caregivers (family, friends)	7
Nurses	12
Physicians	9
Others	4

### Primary Outcome: Effectiveness of eHealth Interventions in Palliative Care

The results for the first study question—efficacy of eHealth system for palliative care—are shown in Table 4. The eight studies describing the effects of eHealth interventions reported being effective with regards to the following aspects of palliative care: improvement of quality of care, improved communication, reduction of documentation effort, and reduction of costs.

A high level of user satisfaction was reported (eg, [10,15,18]). The only article reporting quantitative effects of an eHealth intervention was a nonexperimental study published by Maudlin et al. This was a time-series study in which the authors compared health care utilization before and after implementing the system, which consisted of a combination of text messaging and videophones. According to [15], the number of hospital admissions decreased by 66%, the number of emergency room visits by 19%, and the number of bed days by 77% after introducing the text messaging and videophone devices.

**Table 4:** Results describing efficacy of eHealth system for palliative care.

Author, year	Results
Kallen 2012 [10]	Improved contact with caregivers; better quality of care
Koczwara 2010 [11]	Curriculum influences practice of health professionals
Lind 2004 [12]	Improved contact with caregivers; better quality of care
Lind 2008 [13]	Improved contact with caregivers; better quality of care
Maudlin 2006 [15]	Lower number of hospitalizations, emergency room visits, and bed days; reduced costs
McCall 2008 [16]	Helpful for patients; useful or detecting symptoms earlier
Paré 2009 [18]	Reduction of documentation efforts; more time for direct care
Walker 2006 [23]	28 patients prefer PDA (personal digital assistant) questionnaires; 10 patients prefer paper; 16 had no preference.

### Secondary Outcome: Information Needs in Palliative Care

**Figure 2:**
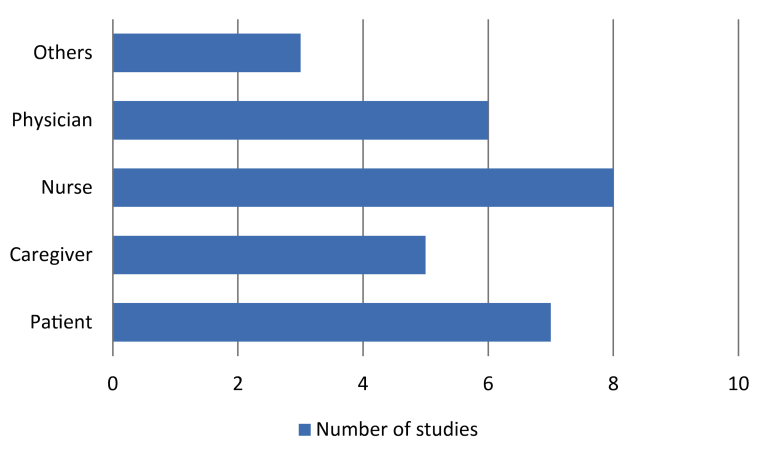
Palliative care roles covered in 11 studies included for information needs.

Table 5 shows the information needs of participants in the palliative care process as reported in the included papers. The most prevalent information need that we identified was information related to pain management, such as recommended drug combinations and dosages. Further questions dealt with the management of other palliative symptoms and care in general. Figure 2 shows how the patient, caregiver, nurse physician, and other roles are represented in the included studies. As shown in Table 5, some studies covered multiple roles. Despite their key role in palliative care, informal caregivers were infrequently included in these studies. Some studies reported that users of the respective system (mostly telephone-based interventions) needed reassurance on the treatment they intended to perform or medication dosage.

**Table 5:** Results for users’ needs.

Author, year	User needs	Participants
Coleman 2005 [25]	Information on medical, experimental, and alternative treatments; information on cancer-related symptoms, prognosis, and end-of-life care	Patients, informal caregivers
Grant 2011 [9]	Information on diseases and care management	Patients
Linklater 2009 [14]	Support with pain management, support with other symptoms	Physicians, family physicians/general practitioners, nurses, patients, and caregivers
Maudlin 2006 [15]	Patients need to feel connected to themselves and others	Patients, informal caregivers, nurses
McCall 2008 [16]	Patients want to express their feelings and reception of their status, patients feel looked after because someone is reading their questionnaire	Patients, nurses, physicians
van Heest 2009 [17]	Consultation on sedation or euthanasia, validation of proposed clinical actions	Physicians, nurses, pharmacists, and specialists
Phillips 2008 [19]	Reassurance on medication usage, support with symptom management, anxiety, death	Patients, informal caregivers, nurses, physicians
Ridley 2008 [20]	Assistance in pain management, support with gastrointestinal concerns, psychosocial, and ethical questions	Nurses, physicians, pharmacists
Roberts 2007 [21]	Support with questions related to care, access to health records	Patients, informal caregivers
Teunissen 2007 [22]	Information about pain, delirium, nausea, and other symptoms	Nurses, physicians, pharmacologist
Wilkes 2004 [24]	Reassurance with specific care questions	Nurses, physicians

## Discussion

### Principal Findings

The literature review resulted in the inclusion of 17 primary studies. The fact that none of these studies were randomized controlled trials and they did not describe patient-relevant clinical outcomes highlights the current need to formally evaluate the effectiveness of these technologies. As far as efficacy of the interventions is concerned, some studies reported positive results in terms of quality of care, communication, and cost development, but since they were all observational or quasi-experimental studies, risk of bias is significant. In addition, IT was used in rather conservative ways throughout the studies. Often, IT was used to mimic analog processes, like filling in questionnaires or simplifying documentation tasks [13,16,18]. The digitalization of analog procedures is probably beneficial for participants in palliative care. However, it seems likely that the application of more advanced information and communication technology developments might lead to better results. For example, communication-centered technologies based on the Web, such as social networking sites, may give patients and caregivers an increased feeling of connectedness; applications that provide interactive learning experiences may help empower patients and caregivers. The proliferation of smartphones and ubiquitous sensors, on the other hand, could help address a broader population with eHealth interventions both in terms of usability and Internet coverage.

To further specify possible fields where knowledge gaps might exist, it is helpful to consider the results of our secondary research question regarding information needs in palliative care. Of the 11 studies that addressed that question, only three reported on an eHealth intervention. Most of the studies were based on synchronous communication over the phone—an approach with limited scalability since it requires the concurrent participation of individuals at an elevated cost [28]. Despite this, the results of these studies are valuable sources to determine the information needs of the roles participating in the palliative care process.

In terms of user needs, the most frequent issue was knowledge about pain management. This need was prevalent not only with patients and informal caregivers, but also health care professionals not specialized in palliative care. Although this finding is not surprising, it provides valuable information on how other technology-based interventions, such as pain monitoring using mobile devices or infusion pumps for analgesics, could be used in the future. There is also evidence that communication itself was an important factor for many users of phone support systems. However, as the results of [16] suggest, verbal communication was not necessarily essential to induce a feeling of connectedness in users of an eHealth system. The possibility to communicate about one’s feelings and problems and the knowledge that someone might read it can be beneficial by itself.

As shown in Figure 2, a great majority of studies are covering information needs reported on health care professionals and patients. Only five studies provided data on the information need of informal caregivers involved in the palliative care process. Considering the high burden experienced by informal caregivers and the high responsibility role they play in many societies, this seems to be a noteworthy shortcoming in current literature.

This systematic review also highlights the lack of information about the use of eHealth for palliative care in developing countries. With all but one study being conducted in high-income countries, information to understand the efficacy of these systems in different economic settings is lacking. This is particularly urgent given the great and accelerating penetration of information technologies, especially mobile phone and Internet connections in developing countries, which is creating large numbers of potential users that could benefit from well-designed systems to support health in general and palliative care in particular. The availability of effective ways to communicate with patients and caregivers, along with effective eHealth interventions or applications, might significantly improve the availability of palliative care especially in underserved populations.

### Limitations of the Review

The studies considered for this research were peer-reviewed publications identified from three scientific databases. The inclusion of additional data sources might have returned additional publications including gray literature, especially concerning the question on information needs. Furthermore, the database queries were narrowed down to eHealth applications. As a consequence, information needs were returned only if they were reported in the context of such applications. Although we included reports on systems based on synchronous communication, there still might be publications covering information needs in different contexts of care or in studies not involving eHealth interventions.

### Future Research Priorities

Future research in the domain of eHealth for palliative care should first concentrate on studying information needs of the diverse roles involved in palliative care. Special focus should be given to informal caregivers, since they carry a major burden of delivering care but are often neglected when new eHealth applications are designed. Research on information needs should lead to data that have a finer granularity, for example, compared to the rather coarse need for information on pain management that we identified. To avoid bias in these investigations, the number of individuals contributing their information needs should be appropriately large.

The information needs should be the basis for developing new eHealth systems targeting the different roles. To validate the approach, this domain needs high-quality randomized controlled trials to formally establish the effectiveness of these interventions.

## Multimedia Appendix 1

Excluded studies.

## Abbreviations

IT: information technology
LILACS: Literature in the Health Sciences in Latin America and the Caribbean
PDA: personal digital assistant
PRISMA: Preferred Reporting Items for Systematic Reviews and Meta-analyses
