# Supplementary material for: Effectiveness of eHealth Interventions and Information Needs in Palliative Care: A Systematic Literature Review
Source: J Med Internet Res. 2014 Mar 7;16(3):e72. doi: 10.2196/jmir.2812 (PMC3961802; doi:10.2196/jmir.2812)
Supplement: Supplementary file 1 [file jmir_v16i3e72_app1.pdf]

TITLE	key	Notes
Patient self-reporting in palliative care using information technology: Yes, there is hope!	[1]	Editorial
Pain and palliative care pharmacotherapy literature summaries and analyses	[2]	collection of literature summaries
Daily life as an Australian medical oncologist	[3]	not specifically on palliative care
Outpatient palliative care: Does it matter?	[4]	conference abstract
Future planning to upgrade the pediatric oncology service in the Baghdad children welfare teaching hospital	[5]	Pediatric
Annual convention: Paramedics and emergency doctors of air rescue service met in Wurzburg	[6]	no ehealth interverntion and no users' needs
Triangulation analysis of tele-palliative care implementation in a rural community area in Japan	[7]	no ehealth intervention, no users' needs
Factors associated with attrition from a randomized controlled trial of meaning-centered group psychotherapy for patients with advanced cancer	[8]	treatment evaluation (psychotherapy)
[The palliative care day hospital mobile team]	[9]	French
[Supporting caregivers in the palliative care unit (interview by Severine Coste)]	[10]	French
Palliative care intervention for choice and use of opioids in the last hours of life	[11]	no ehealth interverntion and no users' needs
Effects of a palliative care intervention on clinical outcomes in patients with advanced cancer: The project ENABLE II randomized controlled trial	[12]	no ehealth interverntion and no information needs
Nursing diagnoses recorded in palliative care documentation. A systematic review	[13]	review on nursing diagnoses related to palliative care
Adopting and introducing new technology to improve patient care: a wedding of clinicians and informatics specialists	[14]	no eHealth
The use of modelling to evaluate new drugs for patients with a chronic condition: The case of antibodies against tumour necrosis factor in rheumatoid arthritis	[15]	report on cancer treatment, not palliative care itself
Online courses for nurses working in palliative care	[16]	report on nursing education, not palliatibe care as such, no users' needs
The veterans health administration system of care for mild traumatic brain injury: Costs, benefits, and controversies	[17]	report on Polytrauma System of Care, not palliative care itself
Paediatric palliative home care with Internet-based video-phones: lessons learnt	[18]	pediatric
Using videotelephony to support paediatric oncology-related palliative care in the home: from abandoned RCT to acceptability study	[19]	pediatric

Improving access to palliative care through an innovative quality improvement initiative: An opportunity for pay-for-performance	[20]	paper on how to increase access to palliative care, no ehealth or users' needs
[The helping relationship. Collaboration between the nurses of a palliative care mobile unit and the nurses of the clinical services]	[21]	French
[Specific missions for the mobile palliative care support teams]	[22]	French
A rapid response veteran affairs outpatient palliative care consult team (316-B)	[23]	conference abstract
Vaginal and pelvic recurrences in stage I and II endometrial carcinoma - Survival and prognostic factors	[24]	report on prognostic factors in cancer
Radiotherapy and chemotherapy in patients with primary invasive vaginal carcinoma	[25]	treatment report
Renewing primary care: Lessons learned from the Spanish health care system	[26]	vision paper on health system development, no individuals' information needs
Oncology's transitional generation: Charting the way to integrated, efficient, and cost-effective delivery systems	[27]	Commentary
[Development of palliative care in the Vaud canton. 3 mobile teams are in place]	[28]	French
Home telemedicine for paediatric palliative care	[29]	Pediatric
Studying the effectiveness of palliative care [1]	[30]	Letter
A framework for measuring quality in the emergency department	[31]	Commentary
Introducing ‘Palcall’: an innovative out-of-hours telephone service led by hospice nurses.	[32]	Neither user needs nor efficacy
Translating research into practice: Reducing gastrostomy tube placement in advanced dementia patients (711)	[33]	conference abstract
A pediatric oncology nursing outreach program	[34]	Pediatric
The role of radiofrequency ablation in the treatment of primary and mestastatic tumours of the liver: Initial lessons learned	[35]	no palliative care as such
Development of terminology subsets using ICNP(registered trademark)	[36]	not on palliative care as such but on nursing terminology
[Activities of a nurse of a palliative mobile unit]	[37]	French
Single-ventricle palliation for high-risk neonates: Examining the feasibility of an automated home monitoring system after stage i palliation	[38]	pediatric, no palliative care

Transitions in care during a palliative care program: Distribution and associated factors	[39]	no ehealth, no users' needs
[Palliative care by a mobile team: objectives and missions, qualities and difficulties]	[40]	French
Patients at highest risk for venous thromboembolism are not receiving specific discharge instructions warning for signs and symptoms of VTE: A double setback	[41]	conference abstract
Palliative treatment of esophageal carcinoma	[42]	treatment report, not palliative care
Effectiveness of a geriatric palliative care consult on pain management and advance directives among older adults	[43]	conference abstract
Making an impact on 30 day heart failure readmissions: A comprehensive and collaborative effort begins	[44]	conference abstract
The feasibility of telephone follow-up led by a radiation therapist: Experience in a multidisciplinary bone metastases clinic	[45]	synchronous communication, no palliative care
Colorectal stenting in large bowel obstruction: Our experience	[46]	conference abstract
Ontario pharmacists practicing in family health teams and the patient-centered medical home	[47]	no palliative care as such
Bridging the distance: a prospective tele-oncology study in Northern Norway	[48]	No palliative care
The impact of a modular HIV/AIDS palliative care education programme in rural Uganda	[49]	no ehealth, no users' needs
Study protocol: Optimization of complex palliative care at home via telemedicine. A cluster randomized controlled trial	[50]	synchroneous communication, no users' needs
[Mobile team of palliative care. Role of the nurse in relation to hospital nurses]	[51]	French
Organizing palliative care for rural populations: A systematic review of the evidence	[52]	no ehealth intervention, no users'needs
Patients' perceptions of services and preferences for care in amyotrophic lateral sclerosis: A review	[53]	no ehealth intervention, no users' needs
Ambulatory oncology consulting team: A bridge between the oncology department and staff in the community health care in the county of Stockholm, Sweden	[54]	no eHealth
Journal of Medical Imaging and Radiation Sciences: Message from the Editor	[55]	Editorial
Recognition and management of acute medication poisoning	[56]	no palliative care as such
[Lois or the end of life of a child suffering from cancer]	[57]	French

Amoxicillin for acute rhinosinusitis: A randomized controlled trial	[58]	Medication trial
[Mobile palliative and supportive care unit]	[59]	French
Stora luckor i journaler vid vard i livets slutskede	[60]	article in Swedisch
Thyroid cancer: possible role of telemedicine	[61]	synchronous communication, no palliative care
Patients' opinion of weekend cover by palliative care general practitioners	[62]	report on care organisation during weekends
Why, How, What, and nullSo What!null developing clinical informatics tools for determined skeptics	[63]	conference abstract
A narrative literature review of the evidence regarding the economic impact of avoidable hospitalizations amongst palliative care patients in the UK	[64]	no ehealth intervention, no users'needs
Specialty services for children with special health care needs: Supplement not supplant the medical home	[65]	pediatric
Which cancer patients are referred to hospital at home for palliative care?	[66]	study on treatment decisions, no users' needs
In this issue	[67]	Editorial
Supportive care assessment: Patient preferences for disclosure of palliative care information	[68]	conference abstract
Out-of-hours prescribing: A survey of current practice in the UK	[69]	report on prescription practice, not palliative care itself
Preliminary analysis of midlevel practitioners on pain and health-related quality of life and function for a palliative care service at a comprehensive cancer center	[70]	letter to the editor
Hospices' Preparation and Practices for Quality Measurement	[71]	no ehealth intervention, study on quality of care
Technical data evaluation of a palliative care web-based documentation system	[72]	technical evaluation, no efficacy, no users' needs
Analysis of the suitability of 'video-visits' for palliative home care: implications for practice	[73]	synchroneous communication, no users' needs
Informatics-enabled behavioral medicine in oncology	[74]	no palliative care as such
Improving medication reconciliation in an outpatient palliative medicine clinic: A quality improvement study	[75]	study on medication diescrepancies
Expert consensus on diagnostic criteria and tertiary service requirements for bronchiectasis	[76]	treatment evaluation
A history of patient education by health professionals in Europe and North America: from authority to shared decision making education	[77]	Historic overview, no original research

Knowledge and Attitude toward Hospice Palliative Care among Community-Dwelling Aged Taiwanese-Analysis of Related Factors	[78]	no ehealth intervention, no users'needs
Surgical palliative care in Haiti	[79]	no ehealth intervention or information needs
Videotelephony: An innovative mode of palliative care service delivery in regional and remote areas	[80]	Pediatric
Heart failure management programmes in Europe	[81]	study on heart failure management, not palliative care
Using the internet to improve the study and management of pain: Could the health system meet our expectations?	[82]	report on pain management, not palliative care as such
An approach for incorporating advanced care planning into heart failure specialty care	[83]	conference abstract
Pelvic reirradiation for recurrent or new primary gynecologic malignancies	[84]	conference abstract
UK telehealth initiatives in palliative care: A review	[85]	synchronous communication, no users' needs
Using technology in palliative care - A reality	[86]	conference abstract
Use of Norethindrone Acetate Alone for Postoperative Suppression of Endometriosis Symptoms	[87]	pediatric
From skype to smart phones: Uses and limits of telehealth in palliative care	[88]	Conference abstract
Empowering local people to run a mobile palliative care service	[89]	no research report
Who says medical education hasn't changed?	[90]	video file
e-Health in pediatric palliative care	[91]	Pediatric
Information seeking behaviors of parents whose children have life-threatening illnesses	[92]	Pediatric
Does evidence-based medicine really reduce costs?	[93]	not on palliative care as such
Spiritual issues in palliative care consultations in the Netherlands	[94]	report on spiritual issues, not an ehealth intervention or information needs.
Videophones for the delivery of home healthcare in oncology	[95]	synchroneous video-telephony, no users' needs
[Palliative care mobile team at a Parisian university hospital]	[96]	French
[What is expected of psychologists in palliative care mobile teams? Their role and missions]	[97]	French
Comparison of uncovered stent with covered stent for treatment of malignant colorectal obstruction{A figure is presented}	[98]	treatment evaluation, no palliative care
Poor Effect of Family Practice Physician Training at the Organizational Level in Long-Term Care Facilities in Flanders, Belgium	[99]	letter to the editor

Communicating with children and families: From everyday interactions to skill in conveying distressing information	[100]	Pediatric
Feasibility of symptom and quality-of-life (QOL) assessment in a VA network-based palliative care (PC) program	[101]	conference abstract
Out-of-hours palliative care advice line	[102]	letter to the editor
Use of radiation treatment units in breast cancer. Changes in the last 15 years	[103]	overview articel on radio therapy, not palliative care
Is it reliable to use cellular phones for symptom assessment in palliative care? Report on a study in patients with advanced cancer	[104]	letter to the editor
Using telehealth technology to support CME in end-of-life care for community physicians in Ontario	[105]	no ehealth intervention, no users' needs
Pediatric palliative care and eHealth: Opportunities for patient-centered care	[106]	Pediatric
Analytic review: Mass-casualty incidents: How does an ICU prepare?	[107]	No palliative care
Telemedicine is crucial for improving access to specialist renal care and management of renal disease in remote/rural locations	[108]	no palliative care
Validation study of an end-of-life questionnaire from the Swedish Register of Palliative Care	[109]	no ehealth intervention, no users' needs
The radiologist as a palliative care subspecialist: Providing symptom relief when cure is not possible	[110]	no ehealth intervention, no users' needs
International collaborations in cancer control and the Third International Cancer Control Congress	[111]	rreort on cancer control in general
New developments in the detection and treatment of depression in cancer settings	[112]	report on depression with cancer patients
Use of ISDN video-phones for clients receiving palliative and antenatal home care	[113]	synchroneous video-telephony
Home videoconferencing for patients with severe congential heart disease following discharge	[114]	Pediatric
Psychosocial supportive care: An effective and integrated team approach	[115]	no ehealth intervention, no users' information needs
Using electronic triggers to identify patients at risk for diagnostic delays in prostate cancer	[116]	no palliative care
Telemedicine in radiation oncology: Challenges and opportunities	[117]	synchronous comunication
Palliative chemotherapy during the last month of life	[118]	no ehealth intervention, no users' needs
Entering our fourth decade	[119]	Pediatric

News from the 40th European symposium on clinical pharmacy; 18-21 October 2011; Dublin	[120]	conference report
A university oncology department and a remote palliative care unit linked together by email and videoconferencing	[121]	synchroneous communication
Dr Shipman's last legacy: E-surveillance of the medical profession	[122]	editorial
Mortality within 30 days of chemotherapy: A clinical governance benchmarking issue for oncology patients	[123]	statistics on mortality, not palliatve care as such
Evaluation of rebound ascites following discontinuation of bevacizumab in recurrent ovarian cancer	[124]	conference abstract
The mobile phone as a tool in improving cancer care in Nigeria	[125]	report on cancer care, not palliative care
Family medicine in 2018	[126]	no palliative care
Osteoradionecrosis of the Mandible: Treatment Outcomes and Factors Influencing the Progress of Osteoradionecrosis	[127]	report on cancer treatment, not palliative care
Retrospective analysis of 191 patients with advanced hepatocellular carcinoma treated with transarterial chemoembolisation (TACE): Validation of the established and construction of an improved HCC-staging-system	[128]	report on cancer treatment, not palliative care
Home care for advanced cancer: Results and challenges	[129]	no eHealth intervention, no user needs
Palliative cancer patients and their families on the Internet: Motivation and impact	[130]	series of case studies, but unclear if cases are real or fictional
Palliative care on the Net: an online survey of health care professionals	[131]	general description of Internet usage, not related to palliative care
Analysis of the activity of a palliative care support team	[132]	no EHealth
Older rural australian men with prostate cancer: Mapping their patterns of care	[133]	conference abstract
Health behaviour interventions for cancer survivors: An overview of the evidence and contemporary Australian trials	[134]	health education for cancer survivors
nullI'm afraid! what happens?null EPR and decision making	[135]	conference abstract
Logistic aspects in emergency care of the elderly	[136]	emergency care, not palliative
The challenges of building and sustaining a pediatric neuro-oncology program in a developing country	[137]	Pediatric
Implementation of the screening for Palliative Care Needs in the Emergency Department (SPEED) instrument in two emergency departments	[138]	conference abstract
Nurse Practitioner led Palliative Care Program	[139]	conference abstract
[Palliative care hospital units]	[140]	French
Using videoconferencing in palliative care	[141]	syncchroneous communication

Videoconferencing and palliative care	[142]	synchroneous communication, no users' needs
[Bretonneau mobile team, a bridge between the hospital and the community]	[143]	French
Combined interventional radiological therapy and radiotherapeutic treatment of patients with carcinomas of the extrahepatic biliary tract	[144]	Oncological treatment evaluation, no palliative care
A coping and communication support intervention tailored to older patients diagnosed with late-stage cancer	[145]	No ehealth
Telemedicine across the ages	[146]	report on symmetric communication, no users' needs
What kind of requests do healthcare professionals make of a telephone out of hours specialist palliative care advice service? The experience of one hospice over a year [3]	[147]	Letter to editor
Telephone consultation in palliative care in remote thar desert of India: 3 Years of experience	[148]	conference abstract
Telemedicine in community-based palliative care: evaluation of a videolink teleconference project	[149]	synchroneous communication, no users' needs
In vivo dosimetry in electron beam teletherapy using electron paramagnetic resonance in L-alanine	[150]	report on treatment method, not palliative care
Out-of-hours palliative care provided by GP co-operatives: Availability, content and effect of transferred information	[151]	no ehealth intervention, no users' needs
GI-cancer patients education: Status of cultural economical and social perceptions in developing nations	[152]	Education on cancer, no palliative care itself
MHWD Telepsychiatry Project 2004	[153]	no palliative care
Life after the women's health initiative: Evaluation of postmenopausal symptoms and use of alternative therapies after discontinuation of hormone therapy	[154]	no palliative care
Telemedicine as a tool for evaluation of retinopathy of prematurity	[155]	no palliative care
Quality-of-care implications of improving physician communication through a Web-based tool	[156]	conference abstract
Re-irradiation of recurrent and/or persistent squamous cell carcinoma of head and neck region	[157]	treatment report
Patient safety incidents in home hospice care: A qualitative study of interdisciplinary hospice team members	[158]	report on patient safety
Oncology patient-centered medical home and accountable cancer care	[159]	no ehealth intervention, no users' needs
[Mobile team for palliative care in Geneva]	[160]	French

Rationale, design, and implementation protocol of the Dutch clinical practice guideline pain in patients with cancer: a cluster randomised controlled trial with Short Message Service (SMS) and Interactive Voice Response (IVR)	[161]	conference abstract
Early palliative care for patients with metastatic non-small-cell lung cancer	[162]	no ehealth intervention, no users' needs
Longitudinal perceptions of prognosis and goals of therapy in patients with metastatic non-small-cell lung cancer: Results of a randomized study of early palliative care	[163]	no ehealth intervention, no users' needs
"You can't always get what you want" - Or can you?	[164]	commentary
Screening for psychological distress in palliative care: performance of touch screen questionnaires compared with semistructured psychiatric interview.	[165]	VAlidation of a touch-screen survey. Not effectiveness or users' needs
Duodenal stents are associated with more durable patency as compared to percutaneous endoscopic gastrojejunostomy in the palliation of malignant gastric outlet obstruction	[166]	no ehealth intervention, no users' needs
Self-expanding metal stents (SEMS) for patients with advanced Esophageal cancer in Malawi: An effective palliative treatment	[167]	report on treatment, not palliative care as such
Marmara Medical Journal: From the editor	[168]	Editorial
[Cancer pain, a palliative care team approach]	[169]	French
Tele-practice guidelines for the symptom management of children undergoing cancer treatment	[170]	Pediatric
The effectiveness of palliative care education delivered by videoconferencing compared with face-to-face delivery.	[171]	videoconferncing, no user needs
The Long-term Efficacy of Pneumatic Dilatation and Heller Myotomy for the Treatment of Achalasia	[172]	no palliative care
Home Oncology Medical Extension (H.O.M.E.) - an effective alternative to hospital care	[173]	no eHealth intervention
Multidisciplinary longitudinal approach to patients with high admission rates	[174]	conference abstract
Immediate or delayed palliative teleradiotherapy patients with advanced non small cell lung cancer?	[175]	palliative teleradiotherapy, not palliative care
Improving outcomes for people with progressive cancer: Interrupted time series trial of a needs assessment intervention	[176]	no eHealth intervention, no users' information needs
Chronic heart failure: promoting quality of life	[177]	no EHealth

Incorporating goals-of-care conversations into hospital admissions at a system-wide level: Policy and educational strategies (306)	[178]	conference abstract
Reflexology for symptom relief in patients with cancer	[179]	no ehealth, no users' needs
Family discord and proxy decision makers' end-of-life treatment decisions	[180]	evaluation of treatment decision (before palliative care)
Systems for the management of respiratory disease in primary care - an international series: United Kingdom	[181]	report on health care system
Advance care planning among the oldest old	[182]	no ehealth, no users' needs
Experiences of 24-hour advice line services: a framework for good practice and meeting NICE guidelines.	[183]	sort of a whitepaper.
Implementing a patient administration system	[184]	report on administration system, no users's needs
The elephant in the room: Negotiating meaningful advance care planning with patients, Families, and clinicians of diverse backgrounds and cultures	[185]	conference abstract

1. Abernethy A, Currow D. Patient self-reporting in palliative care using information technology: Yes, there is hope! Palliative Med 2011; 25(7).

2. Abernethy AP, Zhukovsky D. Pain and palliative care pharmacotherapy literature summaries and analyses. J Pain Palliat Care Pharmacother 2012; 26(1).

3. Adams D, Marx G. Daily life as an Australian medical oncologist. J. Oncol. Pract 2010; 6(3).

4. Albanese T, Radwany S, Taggart C, Chua DJ. Outpatient palliative care: Does it matter? J. Pain Symptom Manage 2012; 43(2).

5. Al-Hadad S, Al-Jadiry MF. Future planning to upgrade the pediatric oncology service in the Baghdad children welfare teaching hospital. J. Pediatr. Hematol. Oncol 2012; 34(SUPPL. 1).

6. Annual convention: Paramedics and emergency doctors of air rescue service met in Wurzburg. Notarzt 2008; 24(4):146.

7. Aoki N, Ohta S, Yamamoto H, Kikuchi N, Dunn K. Triangulation analysis of tele-palliative care implementation in a rural community area in Japan. Telemedicine Journal and e-Health 2006; 12(6):655–62.

8. Applebaum AJ, Lichtenthal WG, Pessin HA, Radomski JN, Simay Gokbayrak N, Katz AM et al. Factors associated with attrition from a randomized controlled trial of meaning-centered group psychotherapy for patients with advanced cancer. Psycho-Oncology.

9. Aubert L, Loureiro E. [The palliative care day hospital mobile team]. Soins; (758):43–4.

10. Baeza C. [Supporting caregivers in the palliative care unit (interview by Severine Coste)]. Soins Gerontol; (89):21.

11. Bailey FA, Ferguson L, Williams BR, Woodby LL, Redden DT, Durham RM et al. Palliative care intervention for choice and use of opioids in the last hours of life. J. Gerontol. Ser. A Biol. Sci. Med. Sci 2008; 63(9).

12. Bakitas M, Lyons KD, Hegel MT, Balan S, Brokaw FC, Seville J et al. Effects of a palliative care intervention on clinical outcomes in patients with advanced cancer: The project ENABLE II randomized controlled trial. J. Am. Med. Assoc 2009; 302(7).

13. Barallat Gimeno E, Palomar Naval C, Jimenez Vilchez A, Barbera Cortada J. Nursing diagnoses recorded in palliative care documentation. A systematic review: El registro de los diagnosticos enfermeros en cuidados paliativos. Revision sistematica de la literatura castellana. Med. Paliativa 2010; 17(4).

14. Barnett J, Syme A. Adopting and introducing new technology to improve patient care: a wedding of clinicians and informatics specialists. Stud Health Technol Inform 2009; 143:343–7.

15. Barton P, Jobanputra P, Wilson J, Bryan S, Burls A. The use of modelling to evaluate new drugs for patients with a chronic condition: The case of antibodies against tumour necrosis factor in rheumatoid arthritis. Health Technol. Assess 2004; 8(11).

16. Becker R. Online courses for nurses working in palliative care. European Journal of Palliative Care 2009; 16(2):94–7.

17. Belanger HG, Uomoto JM, Vanderploeg RD. The veterans health administration system of care for mild traumatic brain injury: Costs, benefits, and controversies. J. Head Trauma Rehabil 2009; 24(1).

18. Bensink M, Armfield N, Russell TG, Irving H, Wootton R. Paediatric palliative home care with Internet-based video-phones: lessons learnt. J Telemed Telecare 2004; 10 Suppl 1:10–3.

19. Bensink ME, Armfield NR, Pinkerton R, Irving H, Hallahan AR, Theodoros DG et al. Using videotelephony to support paediatric oncology-related palliative care in the home: from abandoned RCT to acceptability study. Palliat Med 2009; 23(3):228–37.

20. Bernacki RE, Ko DN, Higgins P, Whitlock SN, Cullinan A, Wilson R et al. Improving access to palliative care through an innovative quality improvement initiative: An opportunity for pay-for-performance. J. Palliative Med 2012; 15(2).

21. Bernard MF. [The helping relationship. Collaboration between the nurses of a palliative care mobile unit and the nurses of the clinical services]. Soins Form Pedagog Encadr 1998; (27):46–51.

22. Beuvin M. [Specific missions for the mobile palliative care support teams]. Soins Gerontol 2005; (55):22–4.

23. Blackstone K. A rapid response veteran affairs outpatient palliative care consult team (316-B). J. Pain Symptom Manage 2011; 41(1):198.

24. Blecharz P, Brandys P, Urbanski K, Reinfuss M, Patla A. Vaginal and pelvic recurrences in stage I and II endometrial carcinoma - Survival and prognostic factors. European Journal of Gynaecological Oncology; 32(4):403–7.

25. Blecharz P, Urbanski K, Reinfuss M, Szatkowski W, Jasiowka M. Radiotherapy and chemotherapy in patients with primary invasive vaginal carcinoma. Wspolczesna Onkologia; 14(4):265–9.

26. Borkan J, Eaton CB, Novillo-Ortiz D, Corte PR, Jadad AR. Renewing primary care: Lessons learned from the Spanish health care system. Health Affairs; 29(8):1432–41.

27. Bosserman LD. Oncology's transitional generation: Charting the way to integrated, efficient, and cost-effective delivery systems. Community Oncol 2012; 9(1):2.

28. Bourgeois Y, Jacques C, Khelifa A. [Development of palliative care in the Vaud canton. 3 mobile teams are in place]. Krankenpfl Soins Infirm 2003; 96(5):52–4.

29. Bradford N, Herbert A, Walker R, Pedersen LA, Hallahan A, Irving H et al. Home telemedicine for paediatric palliative care. In: Studies in Health Technology and Informatics. p. 10–9 .

30. Bruera E. Studying the effectiveness of palliative care [1]. JAMA - Journal of the American Medical Association 2008; 300(9):1022.

31. Cameron PA, Schull MJ, Cooke MW. A framework for measuring quality in the emergency department. Emerg. Med. J 2011; 28(9).

32. Campbell C, Harper A, Elliker M. Introducing 'Palcall': an innovative out-of-hours telephone service led by hospice nurses. Int J Palliat Nurs 2005; 11(11):586–90. PMID:16471046.

33. Campbell M, Walch J, Sanna-Gouin K, Colomba S. Translating research into practice: Reducing gastrostomy tube placement in advanced dementia patients (711). J. Pain Symptom Manage 2011; 41(1):278.

34. Casey S, Hamalainen T, Di Monte B, Drybrough K. A pediatric oncology nursing outreach program. Pediatric Blood and Cancer 2009; 53(5):891.

35. Choy PYG, Koea J, McCall J, Holden A, Osbourne M. The role of radiofrequency ablation in the treatment of primary and mestastatic tumours of the liver: Initial lessons learned. New Zealand Med. J 2002; 115(1159).

36. Coenen A, Kim TY. Development of terminology subsets using ICNP(registered trademark). Int. J. Med. Informatics 2010; 79(7).

37. Combes M. [Activities of a nurse of a palliative mobile unit]. Rev Infirm 1996; (1):31–8.

38. Cross R, Steury R, Randall A, Fuska M, Sable C. Single-ventricle palliation for high-risk neonates: Examining the feasibility of an automated home monitoring system after stage i palliation. Future Cardiol 2012; 8(2).

39. D'Angelo D, Mastroianni C, Casale G, Carbonara L, Vellone E, Alvaro R et al. Transitions in care during a palliative care program: Distribution and associated factors. European Journal of Cancer; 47:S318.

40. d'Herouville D, Combes M, Manenti G, Gandiol C, Cereda C. [Palliative care by a mobile team: objectives and missions, qualities and difficulties]. Rev Infirm 1996; (1):26–30.

41. Dabbagh O, Parker WA, Philpot MA, Keegan MB, Bosanquet JP. Patients at highest risk for venous thromboembolism are not receiving specific discharge instructions warning for signs and symptoms of VTE: A double setback. J. Thromb. Haemost 2009; 7(S2):770.

42. Dabrowski A, Skoczylas T, Zinkiewicz K, Bury J, Borkowski A, Wallner G. Palliative treatment of esophageal carcinoma. Polski Merkuriusz Lekarski 2005; 19(114):804–7.

43. Davila A, Garza M, Lee S, Ross J, Sanchez-Reilly S. Effectiveness of a geriatric palliative care consult on pain management and advance directives among older adults. J. Pain Symptom Manage 2010; 39(2):395.

44. Davis MR, Smith CP, Phillips SG, Semones JG. Making an impact on 30 day heart failure readmissions: A comprehensive and collaborative effort begins. Heart Lung J. Acute Crit. Care 2011; 40(4):376.

45. Dixon W, Danielson B, Pituskin E, Fairchild A, Ghosh S. The feasibility of telephone follow-up led by a radiation therapist: Experience in a multidisciplinary bone metastases clinic. Journal of Medical Imaging and Radiation Sciences; 41(4):175–9.

46. Dixon S, Chung D, Anderson EM, Cunningham C, Anthony S, Bratby M et al. Colorectal stenting in large bowel obstruction: Our experience. Cardiovasc. Intervent. Radiol 2011; 34:493.

47. Dolovich L. Ontario pharmacists practicing in family health teams and the patient-centered medical home. Ann. Pharmacother 2012; 46(4).

48. Donnem T, Ervik B, Magnussen K, Andersen S, Pastow D, Andreassen S et al. Bridging the distance: a prospective tele-oncology study in Northern Norway. Supportive Care in Cancer:1–7.

49. Downing J, Kawuma E. The impact of a modular HIV/AIDS palliative care education programme in rural Uganda. Int J Palliat Nurs 2008; 14(11):560–8.

50. Duursma F, Schers HJ, Vissers KC, Hasselaar J. Study protocol: Optimization of complex palliative care at home via telemedicine. A cluster randomized controlled trial. BMC Palliative Care; 10.

51. Elias C. [Mobile team of palliative care. Role of the nurse in relation to hospital nurses]. Soins 1996; (606):46–7.

52. Evans R, Stone D, Elwyn G. Organizing palliative care for rural populations: A systematic review of the evidence. Fam. Pract 2003; 20(3).

53. Foley G, Timonen V, Hardiman O. Patients' perceptions of services and preferences for care in amyotrophic lateral sclerosis: A review. Amyotrophic Lateral Sclerosis; 13(1):11–24.

54. Forsberg C, Rundstrom C, Randen M, Dahlin Y, Veidemann E, Barkfjard S. Ambulatory oncology consulting team: A bridge between the oncology department and staff in the community health care in the county of Stockholm, Sweden. Supportive Care in Cancer; 18:S87.

55. French J. Journal of Medical Imaging and Radiation Sciences: Message from the Editor. Journal of Medical Imaging and Radiation Sciences; 41(4):171–4.

56. Frithsen IL, Simpson WM. Recognition and management of acute medication poisoning. Am. Fam. Phys 2010; 81(3).

57. Furioli J, Le Guehennec J, Desouches MM, Dureuil L, Thebault J. [Lois or the end of life of a child suffering from cancer]. Soins Pediatr Pueric; (260):21–3.

58. Garbutt JM, Banister C, Spitznagel E, Piccirillo JF. Amoxicillin for acute rhinosinusitis: A randomized controlled trial. JAMA - Journal of the American Medical Association; 307(7):685–92.

59. Ghiglone B, Cholet V, Calcagno B, Llopet C. [Mobile palliative and supportive care unit]. Soins; (754 Suppl):S12-3.

60. Gholiha A, Fransson G, Furst CJ, Heedman P, Lundstrom S, Axelsson B. Stora luckor i journaler vid vard i livets slutskede. Lakartidningen 2011; 108(16).

61. Gibelli G, Gibelli B, Nani F. Thyroid cancer: possible role of telemedicine. Acta otorhinolaryngologica Italica : organo ufficiale della SocietÃ italiana di otorinolaringologia e chirurgia cervico-facciale 2008; 28(6):281–6.

62. Giesen P, Veldhoven C, Vlaar N, Borghuis M, Koetsenruijter J, Verheggen S. Patients' opinion of weekend cover by palliative care general practitioners. Huisarts en Wetenschap; 54(12):646–9.

63. Goebel J, Ahluwalia S, Chong K, Lorenz KA, Walling A. Why, How, What, and "So What!" Developing Clinical Informatics Tools for Determined Skeptics. J. Pain Symptom Manage 2012; 43(2):403.

64. Gott M, Ward S, Gardiner C, Cobb M, Ingleton C. A narrative literature review of the evidence regarding the economic impact of avoidable hospitalizations amongst palliative care patients in the UK. Prog. Palliative Care 2011; 19(6).

65. Graham RJ. Specialty services for children with special health care needs: Supplement not supplant the medical home. Archives of Disease in Childhood 2008; 93(1):2–4.

66. Grande GE, McKerral A, Todd CJ. Which cancer patients are referred to hospital at home for palliative care? Palliative Med 2002; 16(2).

67. Gray BH. In this issue. Milbank Q 2011; 89(3).

68. Gregorio SW, Gustin J, Coller K, Taylor R. Supportive care assessment: Patient preferences for disclosure of palliative care information. J. Pain Symptom Manage 2010; 39(2).

69. Gregory A, Todd J, Wanklyn S. Out-of-hours prescribing: A survey of current practice in the UK. Palliative Medicine 2007; 21(7):575–80.

70. Hansen PR, Ma JD, Atayee RS. Preliminary analysis of midlevel practitioners on pain and health-related quality of life and function for a palliative care service at a comprehensive cancer center. J. Palliative Med 2012; 15(4).

71. Hanson LC, Schenck AP, Rokoske FS, Abernethy AP, Kutner JS, Spence C et al. Hospices' Preparation and Practices for Quality Measurement. J. Pain Symptom Manage 2010; 39(1).

72. Hartz T, Bruntrup R, Uckert F. Technical data evaluation of a palliative care web-based documentation system. In: Studies in Health Technology and Informatics : Stud. Health Technol. Informatics; 2011 .

73. Hebert MA, Paquin MJ, Whitten L, Cai P. Analysis of the suitability of 'video-visits' for palliative home care: implications for practice. Journal of telemedicine and telecare 2007; 13(2):74–8.

74. Hesse BW, Suls JM. Informatics-enabled behavioral medicine in oncology. Cancer J 2011; 17(4).

75. Hoeksema L, Montagnini M, Zaleon C. Improving medication reconciliation in an outpatient palliative medicine clinic: A quality improvement study. J. Pain Symptom Manage 2012; 43(2).

76. Holme J, Bianchi S, Clifton I, De-Soyza A, Edenborough F, Peckham D et al. Expert consensus on diagnostic criteria and tertiary service requirements for bronchiectasis. Thorax; 66:A50.

77. Hoving C, Visser A, Mullen PD, van den Borne B. A history of patient education by health professionals in Europe and North America: from authority to shared decision making education. Patient Educ Couns; 78(3):275–81.

78. Hsu C, Chen H, Lee S, Tsou M. Knowledge and Attitude toward Hospice Palliative Care among Community-Dwelling Aged Taiwanese-Analysis of Related Factors. Int. J. Gerontol 2012.

79. Huffman JL. Surgical palliative care in Haiti. Surg Clin North Am; 91(2):445-57, x.

80. Irving H, Bensink M, Herbert A, Bradford N. Videotelephony: An innovative mode of palliative care service delivery in regional and remote areas. Pediatric Blood and Cancer; 55(5):948.

81. Jaarsma T, Stromberg A, Geest S de, Fridlund B, Heikkila J, Martensson J et al. Heart failure management programmes in Europe. European Journal of Cardiovascular Nursing 2006; 5(3):197–205.

82. Jadad AR, Bender JL. Using the internet to improve the study and management of pain: Could the health system meet our expectations? Reviews in Analgesia 2006; 9(1):21–9.

83. Jaenicke C, Wagner J, Florea V. An approach for incorporating advanced care planning into heart failure specialty care. J. Card. Fail 2009; 15(6):S121.

84. Jensen CA, Greven KM. Pelvic reirradiation for recurrent or new primary gynecologic malignancies. Brachytherapy; 9:S51.

85. Johnston B. UK telehealth initiatives in palliative care: A review. International Journal of Palliative Nursing; 17(6):301–8.

86. Johnston BM. Using technology in palliative care - A reality. European Journal of Cancer; 47:S5.

87. Kaser DJ, Missmer SA, Berry KF, Laufer MR. Use of Norethindrone Acetate Alone for Postoperative Suppression of Endometriosis Symptoms. J. Pediatr. Adolesc. Gynecol 2012; 25(2).

88. Keeney C, Head B, Pfeifer M. From skype to smart phones: Uses and limits of telehealth in palliative care. Journal of Pain and Symptom Management; 43(2):392–3.

89. Kendall-Carpenter E. Empowering local people to run a mobile palliative care service. Nurs N Z; 17(4):26–7.

90. Kirch DG. Who says medical education hasn't changed? Medgenmed Medscape Gen. Med 2007; 9(3).

91. Knapp C. e-Health in pediatric palliative care. American Journal of Hospice and Palliative Medicine; 27(1):66–73.

92. Knapp C, Madden V, Marcu M, Wang H, Curtis C, Sloyer P et al. Information seeking behaviors of parents whose children have life-threatening illnesses. Pediatric Blood and Cancer; 56(5):805–11.

93. Kolodziej MA. Does evidence-based medicine really reduce costs? Oncology (USA) 2011; 25(3).

94. Kuin A, Deliens L, van Zuylen L, Courtens AM, Vernooij-Dassen MJFJ, van der Linden B et al. Spiritual issues in palliative care consultations in the Netherlands. Palliative Medicine 2006; 20(6):585–92.

95. Laila M, Rialle V, Nicolas L, Duguay C, Franco A. Videophones for the delivery of home healthcare in oncology. In: Studies in Health Technology and Informatics. p. 39–44 .

96. Lassauniere JM. [Palliative care mobile team at a Parisian university hospital]. Rev Med Brux 2002; 23(1):27–30.

97. Laval G, Villard ML, Comandini F. [What is expected of psychologists in palliative care mobile teams? Their role and missions]. Presse Med 2003; 32(15):677–82.

98. Lee KM, Shin SJ, Hwang JC, Cheong JY, Yoo BM, Lee KJ et al. Comparison of uncovered stent with covered stent for treatment of malignant colorectal obstructionA figure is presented. Gastrointestinal Endoscopy 2007; 66(5):931–6.

99. Lepeleire J, Leirman W. Poor Effect of Family Practice Physician Training at the Organizational Level in Long-Term Care Facilities in Flanders, Belgium. J. Am. Med. Dir. Assoc 2006; 7(7):470.

100. Levetown M. Communicating with children and families: From everyday interactions to skill in conveying distressing information. Pediatrics 2008; 121(5):e1441-e1460.

101. Livote E, Chang V, Cortez T, Heyding R, Luhrs C, Penrod J et al. Feasibility of symptom and quality-of-life (QOL) assessment in a VA network-based palliative care (PC) program. J. Pain Symptom Manage 2010; 39(2):386.

102. Lloyd-Williams M. Out-of-hours palliative care advice line. Br J Gen Pract 2001; 51(469):677.

103. Lopez MA, Latiesas XS, Arnalot PF, Bassols ML, Castillejo AR, Galan JL et al. Use of radiation treatment units in breast cancer. Changes in the last 15 years. Clinical and Translational Oncology 2008; 10(1):47–51.

104. Lundstrom S. Is it reliable to use cellular phones for symptom assessment in palliative care? Report on a study in patients with advanced cancer. Journal of Palliative Medicine 2009; 12(12):1087.

105. Lynch J, Weaver L, Hall P, Langlois S, Stunt M, Schroder C et al. Using telehealth technology to support CME in end-of-life care for community physicians in Ontario. Telemed J E Health 2004; 10(1):103–7.

106. Madhavan S, Sanders AE, Chou WYS, Shuster A, Boone KW, Dente MA et al. Pediatric palliative care and eHealth: Opportunities for patient-centered care. American Journal of Preventive Medicine; 40(5 SUPPL. 2):S208-S216.

107. Mahoney EJ, Biffl WL, Cioffi WG. Analytic review: Mass-casualty incidents: How does an ICU prepare? Journal of Intensive Care Medicine 2008; 23(4):219–35.

108. Majoni W. Telemedicine is crucial for improving access to specialist renal care and management of renal disease in remote/rural locations. Internal Medicine Journal; 41:11.

109. Martinsson L, Heedman P, Lundstrom S, Fransson G, Axelsson B. Validation study of an end-of-life questionnaire from the Swedish Register of Palliative Care. Acta Oncol 2011; 50(5).

110. McCullough HK, Bain RM, Clark HP, Requarth JA. The radiologist as a palliative care subspecialist: Providing symptom relief when cure is not possible. Am. J. Roentgenol 2011; 196(2).

111. Micheli A, Sanz N, Mwangi-Powell F, Coleman MP, Neal C, Ullrich A et al. International collaborations in cancer control and the Third International Cancer Control Congress. Tumori 2009; 95(5):579–96.

112. Mitchell AJ. New developments in the detection and treatment of depression in cancer settings. Progress in Neurology and Psychiatry; 15(5):12–20.

113. Miyazaki M, Stuart M, Liu L, Tell S, Stewart M. Use of ISDN video-phones for clients receiving palliative and antenatal home care. Journal of telemedicine and telecare 2003; 9(2):72–7.

114. Morgan GJ, Craig B, Grant B, Sands A, Doherty N, Casey F. Home videoconferencing for patients with severe congential heart disease following discharge. Congenital Heart Disease 2008; 3(5):317–24.

115. Morrison D. Psychosocial supportive care: An effective and integrated team approach. Psycho-Oncology 2009; 18:S5-S6.

116. Murphy DR, Laxmisan A, Reis BA, Thomas EJ, Singh H. Using electronic triggers to identify patients at risk for diagnostic delays in prostate cancer. J. Gen. Intern. Med 2011; 26.

117. Nair MK, Varghese C, Kumar A, Manoj G, Sudhamony S. Telemedicine in radiation oncology: Challenges and opportunities. Journal International Medical Sciences Academy 2005; 18(1):39–41.

118. Nappa U, Lindqvist O, Rasmussen BH, Axelsson B. Palliative chemotherapy during the last month of life. Ann. Oncol 2011; 22(11).

119. Nazarian LF. Entering our fourth decade. Pediatrics in Review 2009; 30(1):3–4.

120. News from the 40th European symposium on clinical pharmacy; 18-21 October 2011; Dublin. Drugs Ther. Perspect 2012; 28(1).

121. Norum J, Jordhoy MS. A university oncology department and a remote palliative care unit linked together by email and videoconferencing. Journal of telemedicine and telecare 2006; 12(2):92–6.

122. Nutt DJ. Dr Shipman's last legacy: E-surveillance of the medical profession. J. Psychopharmacol 2005; 19(5):441.

123. O'Brien MER, Borthwick A, Rigg A, Leary A, Assersohn L, Last K et al. Mortality within 30 days of chemotherapy: A clinical governance benchmarking issue for oncology patients. Br. J. Cancer 2006; 95(12).

124. O'Cearbhaill RE, Zhou Q, Iasonos A, Spriggs D, Aghajanian C, Sabbatini P. Evaluation of rebound ascites following discontinuation of bevacizumab in recurrent ovarian cancer. Ann. Oncol 2010; 21:viii309.

125. Odigie VI, Yusufu LMD, Dawotola DA, Ejagwulu F, Abur P, Mai A et al. The mobile phone as a tool in improving cancer care in Nigeria. Psycho-Oncology 2012; 21(3).

126. Ogle KD, Boule R, Boyd RJ, Brown G, Cervin C, Dawes M et al. Family medicine in 2018: La medecine familiale en 2018. Can. Fam. Phys 2010; 56(4).

127. Oh HK, Chambers MS, Martin JW, Lim HJ, Park HJ. Osteoradionecrosis of the Mandible: Treatment Outcomes and Factors Influencing the Progress of Osteoradionecrosis. Journal of Oral and Maxillofacial Surgery 2009; 67(7):1378–86.

128. Op Den Winkel M, Nagel D, Op Den Winkel P, Sappl J, Straub G, Lamerz R et al. Retrospective analysis of 191 patients with advanced hepatocellular carcinoma treated with transarterial chemoembolisation (TACE): Validation of the established and construction of an improved HCC-staging-system. J. Hepatol 2011; 54:S397.

129. Pannuti F, Tanneberger S. Home care for advanced cancer: Results and challenges. Archive of Oncology; 18(3):79–83.

130. Pereira J, Bruera E, Macmillan K, Kavanagh S. Palliative cancer patients and their families on the Internet: motivation and impact. J Palliat Care 2000; 16(4):13–9. PMID:11965929.

131. Pereira J, Bruera E, Quan H. Palliative care on the net: an online survey of health care professionals. J Palliat Care 2001; 17(1):41–5. PMID:11324184.

132. Perez Durillo FT, Gallego Montalban JA, Jaen Castillo P. Analysis of the activity of a palliative care support team. Medicina Paliativa.

133. Phillips JL, West PA, Harrington G, Gorzynska K, Shakespeare T. Older rural australian men with prostate cancer: Mapping their patterns of care. Asia-Pac. J. Clin. Oncol 2010; 6:179.

134. Pollard A, Eakin E, Vardy J, Hawkes A. Health behaviour interventions for cancer survivors: An overview of the evidence and contemporary Australian trials. Cancer Forum 2009; 33(3):182–6.

135. Porta E, Manenti E, Rizzi B, Luca B, Cattaneo D. ''I'm Afraid! What Happens?'' EPR and Decision Making. Palliative Med 2010; 24(4):S89.

136. Pruckner S, Martin S, Kleinberger T, Madler C, Luiz T. Logistic aspects in emergency care of the elderly. Notfall und Rettungsmedizin; 14(3):197–201.

137. Qaddoumi I. The challenges of building and sustaining a pediatric neuro-oncology program in a developing country. Neuro-Oncology 2009; 11(6):900.

138. Quest TE, Gisondi MA, Engel KG, Emanuel LL, Goldstein CE. Implementation of the screening for Palliative Care Needs in the Emergency Department (SPEED) instrument in two emergency departments. Acad. Emerg. Med 2011; 18(5):S194.

139. Randle M. Nurse Practitioner led Palliative Care Program. J. Palliative Med 2011; 14(3):A4.

140. Rautureau P. [Palliative care hospital units]. Rev Infirm 2008; (143):32–3.

141. Regnard C. Using videoconferencing in palliative care. Palliat Med 2000; 14(6):519–28.

142. Regnard C. Videoconferencing and palliative care. European Journal of Palliative Care 2000; 7(5):168–71.

143. Remy I. [Bretonneau mobile team, a bridge between the hospital and the community]. Soins Gerontol 2007; (64):35–8.

144. Riedmann E, Pichler L. Combined interventional radiological therapy and radiotherapeutic treatment of patients with carcinomas of the extrahepatic biliary tract. Journal fuer Gastroenterologische und Hepatologische Erkrankungen 2005; 3(2):31–5.

145. Rose JH, Radziewicz R, Bowman KF, O'Toole EE. A coping and communication support intervention tailored to older patients diagnosed with late-stage cancer. Clinical Interventions in Aging 2008; 3(1):77–95.

146. Sabesan SS, Nel P, Varma SC. Telemedicine across the ages. Medical Journal of Australia 2009; 190(12):719.

147. Salt S. What kind of requests do healthcare professionals make of a telephone out of hours specialist palliative care advice service? The experience of one hospice over a year [3]. Palliative Medicine 2007; 21(1):61–2.

148. Sanchetee S, Sanchetee S. Telephone consultation in palliative care in remote thar desert of India: 3 Years of experience. Supportive Care in Cancer 2010; 18(3 Supplement):S176.

149. Saysell E, Routley C. Telemedicine in community-based palliative care: evaluation of a videolink teleconference project. International Journal of Palliative Nursing 2003; 9(11):489–95.

150. Schultka K, Ciesielski B, Wysocka B. In vivo dosimetry in electron beam teletherapy using electron paramagnetic resonance in L-alanine. Nowotwory 2005; 55(6):448–51.

151. Schweitzer BPM, Blankenstein N, Deliens L, van der Horst H. Out-of-hours palliative care provided by GP co-operatives: Availability, content and effect of transferred information. BMC Palliative Care 2009; 8.

152. Shankpal P, Vaishali S. GI-cancer patients education: Status of cultural economical and social perceptions in developing nations. Annals of Oncology; 21:vi45.

153. Shearer R, Macaulay S. MHWD Telepsychiatry Project 2004. Healthc. Rev. Online 2005; 9(2).

154. Shrader SP, Ragucci KR. Life after the women's health initiative: Evaluation of postmenopausal symptoms and use of alternative therapies after discontinuation of hormone therapy. Pharmacotherapy 2006; 26(10).

155. Silva RA, Moshfeghi DM. Telemedicine as a tool for evaluation of retinopathy of prematurity. International Ophthalmology Clinics; 51(1):33–48.

156. Silva AP, Bertoni VD, Mulvey TM, Sampaio C. Quality-of-care implications of improving physician communication through a Web-based tool (Teamwork). ASCO Meeting Abstracts 2012; 30(34_suppl):310.

157. Singh K, Shekhar S, Baruah JD, Bahadur AK, Gulati A, Rathi AK. Re-irradiation of recurrent and/or persistent squamous cell carcinoma of head and neck region. Radiotherapy and Oncology; 99:S333.

158. Smucker D, Regan S. Patient safety incidents in home hospice care: A qualitative study of interdisciplinary hospice team members. Journal of Pain and Symptom Management; 43(2):462–3.

159. Sprandio JD. Oncology patient-centered medical home and accountable cancer care. Community Oncol 2010; 7(12).

160. Steiner N, Luchsinger V. [Mobile team for palliative care in Geneva]. Rev Med Suisse Romande 1997; 117(3):249–53.

161. te Boveldt N, Engels Y, Besse K, Vissers K, Vernooij-Dassen M. Rationale, design, and implementation protocol of the Dutch clinical practice guideline pain in patients with cancer: a cluster randomised controlled trial with Short Message Service (SMS) and Interactive Voice Response (IVR). Implement Sci 2011; 6:126. PMID:22142327.

162. Temel JS, Greer JA, Muzikansky A, Gallagher ER, Admane S, Jackson VA et al. Early palliative care for patients with metastatic non-small-cell lung cancer. New Engl. J. Med 2010; 363(8).

163. Temel JS, Greer JA, Admane S, Gallagher ER, Jackson VA, Lynch TJ et al. Longitudinal perceptions of prognosis and goals of therapy in patients with metastatic non-small-cell lung cancer: Results of a randomized study of early palliative care. J. Clin. Oncol 2011; 29(17).

164. Teno JM, Dosa D. "You can't always get what you want" - Or can you? CMAJ 2006; 174(5).

165. Thekkumpurath P, Venkateswaran C, Kumar M, Newsham A, Bennett MI. Screening for psychological distress in palliative care: performance of touch screen questionnaires compared with semistructured psychiatric interview. J Pain Symptom Manage 2009; 38(4):597–605. PMID:19692204.

166. Thlick JC, Strand D, Patrie JT, Gaidhane M, Kahaleh M, Wang AY. Duodenal stents are associated with more durable patency as compared to percutaneous endoscopic gastrojejunostomy in the palliation of malignant gastric outlet obstruction. Gastrointest. Endosc 2011; 73(4):AB242.

167. Thumbs A, Borgstein E, Vigna L, Kingham TP, Kushner AL, Hellberg K et al. Self-expanding metal stents (SEMS) for patients with advanced Esophageal cancer in Malawi: An effective palliative treatment. Journal of Surgical Oncology; 105(4):410–4.

168. Tozun N. Marmara Medical Journal: From the editor. Marmara Medical Journal 2003; 16(2):77–8.

169. Triol I. [Cancer pain, a palliative care team approach]. Rev Infirm 2008; (140):33–5.

170. Tsimicalis A, Courcy MJ de, Di Monte B, Armstrong C, Bambury P, Constantin J et al. Tele-practice guidelines for the symptom management of children undergoing cancer treatment. Pediatric Blood and Cancer; 57(4):541–8.

171. van Boxell P, Anderson K, Regnard C. The effectiveness of palliative care education delivered by videoconferencing compared with face-to-face delivery. Palliat Med 2003; 17(4):344–58. PMID:12822852.

172. Vela MF, Richter JE, Khandwala F, Blackstone EH, Wachsberger D, Baker ME et al. The Long-term Efficacy of Pneumatic Dilatation and Heller Myotomy for the Treatment of Achalasia. Clinical Gastroenterology and Hepatology 2006; 4(5):580–7.

173. Vinciguerra V. Home Oncology Medical Extension (H.O.M.E.) - an effective alternative to hospital care. Prog Clin Biol Res 1983; 120:319–26.

174. Virapongse A, Sullivan E, Ree J, Chun A, Daisley W, Friedrich M et al. Multidisciplinary longitudinal approach to patients with high admission rates. J. Hosp. Med 2012; 7:S136.

175. Walasek T, Reinfuss M, Blecharz P, Jakubowicz J, Skotnicki P, Pluta E. Immediate or delayed palliative teleradiotherapy patients with advanced non small cell lung cancer? Nowotwory 2009; 59(4):283–6.

176. Waller A, Girgis A, Johnson C, Lecathelinais C, Sibbritt D, Forstner D et al. Improving outcomes for people with progressive cancer: Interrupted time series trial of a needs assessment intervention. Journal of Pain and Symptom Management; 43(3):569–81.

177. While A, Kiek F. Chronic heart failure: promoting quality of life. British journal of community nursing 2009; 14(2):54–9.

178. White J, Fromme E. Incorporating goals-of-care conversations into hospital admissions at a system-wide level: Policy and educational strategies (306). J. Pain Symptom Manage 2011; 41(1):183.

179. Wilkinson S, Lockhart K, Gambles M, Storey L. Reflexology for symptom relief in patients with cancer. Cancer Nurs 2008; 31(5).

180. Winter L, Parks SM. Family discord and proxy decision makers' end-of-life treatment decisions. Journal of Palliative Medicine 2008; 11(8):1109–14.

181. Worth A, Pinnock H, Fletcher M, Hoskins G, Levy M, Sheikh A. Systems for the management of respiratory disease in primary care - an international series: United Kingdom. Primary Care Respiratory Journal; 20(1):23–32.

182. Wu P, Lorenz KA, Chodosh J. Advance care planning among the oldest old. J. Palliative Med 2008; 11(2).

183. Yardley SJ, Codling J, Roberts D, O Donnell V, Taylor S. Experiences of 24-hour advice line services: a framework for good practice and meeting NICE guidelines. Int J Palliat Nurs 2009; 15(6):266, 268-71. PMID:19568212.

184. Young G. Implementing a patient administration system. European Journal of Palliative Care 2000; 7(1):26–8.

185. Zhukovsky D, Glajchen M, Robin Lustbader D. The elephant in the room: Negotiating meaningful advance care planning with patients, Families, and clinicians of diverse backgrounds and cultures. J. Pain Symptom Manage 2012; 43(2).
